# Time-lapse mechanical imaging of neural tube closure in live embryo using Brillouin microscopy

**DOI:** 10.1038/s41598-023-27456-z

**Published:** 2023-01-06

**Authors:** Chenchen Handler, Giuliano Scarcelli, Jitao Zhang

**Affiliations:** 1 Fischell Department of Bioengineering, A. James Clark School of Engineering, University of Maryland, College Park, MD 20742 USA; 2 Department of Biomedical Engineering, Wayne State University, Detroit, MI 48201 USA

**Keywords:** Optical imaging, Neurulation, Biomedical engineering, Raman spectroscopy

## Abstract

Neural tube closure (NTC) is a complex process of embryonic development involving molecular, cellular, and biomechanical mechanisms. While the genetic factors and biochemical signaling have been extensively investigated, the role of tissue biomechanics remains mostly unexplored due to the lack of tools. Here, we developed an optical modality that can conduct time-lapse mechanical imaging of neural plate tissue as the embryo is experiencing neurulation. This technique is based on the combination of a confocal Brillouin microscope and a modified *ex ovo* culturing of chick embryo with an on-stage incubator. With this technique, for the first time, we captured the mechanical evolution of the neural plate tissue with live embryos. Specifically, we observed the continuous increase in tissue modulus of the neural plate during NTC for *ex ovo* cultured embryos, which is consistent with the data of *in ovo* culture as well as previous studies. Beyond that, we found that the increase in tissue modulus was highly correlated with the tissue thickening and bending. We foresee this non-contact and label-free technique opening new opportunities to understand the biomechanical mechanisms in embryonic development.

## Introduction

Neural tube closure (NTC) is a central procedure of vertebrate neurulation where the planar neural plate will be elevated and fused to form a hollow neural tube. A failure of this procedure can result in severe neural tube defects, which represent one of the most common human birth defects^1^. Genetic and molecular processes that guide NTC have been extensively studied for many decades^2–4^. On the other hand, biomechanical mechanisms that may be involved in NTC are attracting increasing attention in recent years^5–7^. On the cell and tissue levels, the morphogenesis of the neural tube can be considered as a result of the interaction between the generated force and the mechanical resistance of the embryonic tissue^8,9^: the successful closure of the neural tube requires that the intrinsic force can overcome the opposing tissue tension that relies on its elastic property. As such, the alteration of tissue biomechanics can cause failure of closure and thus malformation of the neural tube^10^. Although the force production and the mechanical change of tissue during the procedure of NTC have been observed in experiments^10–12^, the quantitative contribution of specific biomechanical processes to ensure robust neurulation remains mostly unknown. One of the main reasons is the lack of tools that can map the biomechanics of neural plate tissue in situ and in real time when the embryo is developing.

Many important techniques have been developed to quantify the mechanical properties of embryonic tissue^13^, which can be approximately classified into three categories: (1) contact-based techniques, including atomic force microscopy (AFM)^14,15^ or microcantilever^11,16,17^ based indentations for measuring apparent Young’s modulus in nm to µm scale, micropipette aspiration for measuring tissue tension in µm scale^18^, and tensile test of tissue in ~ mm scale^19^. While the contact-based techniques can provide direct quantification of tissue’s viscoelastic properties at quasi-static or low frequency condition, they need physical access to the sample and need to apply force to deform the sample during measurement. Since neural tube tissue has irregular shape in 3D and is mechanically interconnected, isolate explants are usually required for unambiguous mechanical tests. (2) Bead/droplet-based sensors, including optical/magnetic tweezer^20,21^ and microdroplet^22^. Optical/magnetic tweezer uses force-driven rigid beads (~ µm in diameter) to sense the rheological properties of localized tissue, and microdroplet uses deformable droplets (4–80 µm in diameter) to quantify the tissue stress. These sensors can quantitatively measure the mechanical properties with subcellular or cellular resolution after careful calibration. However, they require injection of beads or droplets into tissue, making them invasive and low throughput. (3) Tissue ablation/dissection. This method uses either an ultrafast pulsed laser beam^10^ or a blade^23^ to dissect a portion of the tissue and evaluate the tissue tension based on the relaxation response. This is an attractive technique because of the simple setup. However, due to the mechanical connection of embryonic tissue in 3D, this method mostly provides global assessment on a relatively large scale (~ 100 µm to ~ mm size). To summarize, existing methods can quantify various aspects of the mechanical properties of cell and tissue with different spatial and temporal scales and have greatly advanced the assessment of embryonic tissue biomechanics. However, due to the technical limitations, the in situ mechanical mapping of the neural plate tissue during the procedure of NTC in live embryos has not been reported.

Confocal Brillouin microscopy is an emerging technique for quantifying the mechanical properties of biological materials^24–26^. Different from conventional mechanical test methods, Brillouin microscopy uses a laser beam to measure the elastic properties of the material. This is based on an optical phenomenon called spontaneous Brillouin light scattering^27^, where the interaction of the incident laser beam and the inherent acoustic phonon within the material will introduce a frequency shift (i.e., Brillouin shift) to the scattered light (see “Methods”). By measuring the Brillouin shift of the scattered light using a customized spectrometer, the elastic longitudinal modulus of the material can be directly quantified. Since Brillouin microscope is designed in a confocal configuration, it can achieve diffraction-limited spatial resolution. In the past several years, we have innovated this technique and demonstrated its feasibility for quantifying the mechanical properties of single cell^28,29^, embryonic tissue^30^, and neural plate^31^ with subcellular resolution and sufficient mechanical sensitivity. As an all-optical technique, Brillouin microscope can measure biomechanics in a non-contact, non-invasive, and label-free manner. Therefore, it could be a promising tool for mapping the elastic properties of neural plate tissue in situ during embryonic development.

In this work, we developed an optical modality that can conduct time-lapse mechanical imaging of NTC in chick embryo. To do so, we integrated a confocal Brillouin microscope with an on-stage incubator for modified *ex ovo* culturing. The *ex ovo* culture ensures the embryo continuously develops over 21 h, covering the complete events of NTC. Comparing with *in ovo* culture where the embryo is developing inside an opaque eggshell, the *ex ovo* culture provides superior optical accessibility and thus allows real-time bright-field and Brillouin imaging during the development of the embryo. We then used Brillouin microscopy to continuously acquire 2D mechanical images of cranial neural plate tissue as the embryo experienced neurulation. With this modality, we observed a distinct increase in the averaged Brillouin shift of the neural plate tissue during the NTC of the *ex ovo* cultured chick embryo, which is consistent with the results of the *in ovo* culturing system. Importantly, we found the increase in tissue modulus on the dorsal–ventral axis was strongly correlated with the thickening of the neural plate as well as the closure angle, indicating the tissue mechanics may be synchronized with the geometric change of the neural plate to achieve a successful closure of the neural tube. Together, this time-lapse mechanical imaging modality can provide new data for understanding the biomechanical mechanisms during embryonic neurulation.

## Results

### Time-lapse mechanical imaging modality allows longitudinal measurement of live embryo

**Figure 1:**
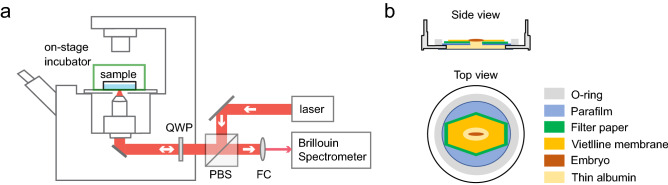
Schematic of the setup. (**a**) Confocal Brillouin microscope with on-stage incubator. *QWP* quarter-wave plate, *PBS* polarized beam splitter, *FC* fiber coupler. (**b**) Carrier dish for *ex ovo* culture. Side view (top) and top view (bottom) displays all components including embryo within the carrier.

Time-lapse 2D mechanical imaging was performed on an inverted Brillouin microscope (Fig. 1a) (see “Methods”). By definition, the Brillouin shift is positively linked to the longitudinal modulus by material properties including refractive index $n$ and density $\rho$. For biological materials, the ratio of refractive index and density $\rho /{n}^{2}$ is found to be approximately constant during physiological processes^24,32^. Therefore, we here used the Brillouin shift to interpret the relative change of longitudinal modulus. In the modified *ex ovo* culture, the embryo was placed into a petri dish with the dorsal side facing down (Fig. 1b). The dish was then placed into the on-stage incubator to sustain the development of the embryo (> 21 h). The time-lapse bright-field images suggest the embryos from *ex ovo* culture have developed with the similar time rate as those from *in ovo* culture (Supplementary Figs. S1–S2).

### *In ovo* cultured embryos show increased Brillouin shift of cranial neural plate against developmental stage

**Figure 2:**
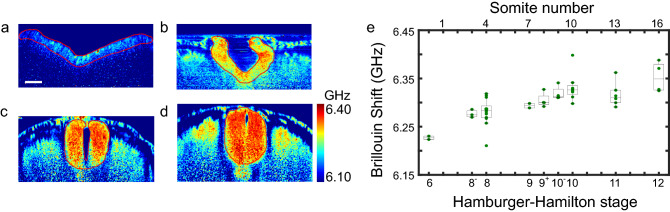
Results of *in ovo* cultured embryos. (**a–d**) Representative Brillouin images of cranial neural tube tissue of four embryos at different HH stages: (**a**) HH 6, (**b**) HH 8, (**c**) HH 11, (**d**) HH 12. Red dashed line outlines the neural plate region. (**e**) Average Brillouin shifts of cranial neural plate of embryos reveal continuous increase in tissue modulus against developmental stage. Number of embryos: 46. Each dot represents the averaged Brillouin shift of one embryo. Scale bar is 50 µm.

To exclude any potential impact of the *ex ovo* culture and the laser illumination on the tissue mechanics of the neural plate, we collected *in ovo* cultured embryos (N = 46) at different Hamburger Hamilton (HH) stages (HH 6 to HH 12) and acquired 2D mechanical images of the cross-section perpendicular to the anterior–posterior axis (close to cranial region). The representative Brillouin images suggest that the neural plate of the embryo at later HH stages has higher Brillouin shift than earlier stages (Fig. 2a–d). We then quantified the average Brillouin shift of the neural plate region at a similar location of anterior–posterior axis for all the collected embryos. We observed that the Brillouin shift of the neural plate showed a distinct increase from HH 6 to HH 9 + and approximately maintained its value afterward (Fig. 2e). The neural plate of latter-stage embryo (i.e., HH 12) has an average Brillouin shift of 6.353 GHz, which is 0.126 GHz higher than that of early-stage embryos (i.e., HH 6), corresponding to ~ 60% increase of Young’s modulus according to the empirical relationship between longitudinal modulus and Young’s modulus obtained from cells^28^ (see “Methods”).

### Time-lapse mechanical imaging of *ex ovo* cultured embryo shows increasing Brillouin shift and thickness of the neural plate during NTC

**Figure 3:**
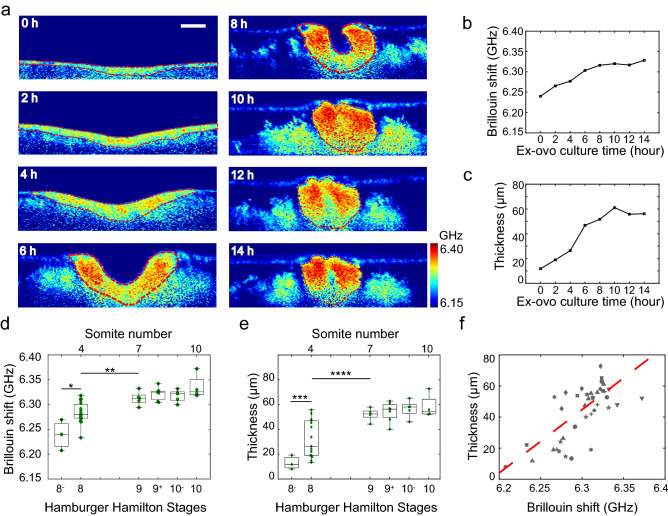
Time-lapse Brillouin imaging of *ex ovo* cultured embryos. (**a**) Time-lapse Brillouin images of a representative embryo for 14 h. (**b**) Average Brillouin shift of neural plate tissue of the embryo in (**a**) is increasing with culturing time. (**c**) Thickness of neural plate tissue of the embryo in (**a**) is increasing with culturing time. (**d**) Increase in Brillouin shift against developmental stage is observed for all the embryos (N = 9). (**e**) Tissue thickening against developmental stage is observed for all the embryos. (**f**) Correlation between the average Brillouin shift and the thickness of neural plate tissue. Symbols represent different embryos. Number of measurements: 39. Two-sample *t* test is used to quantify the statistical significance. *p = 0.008; **p = 0.012; ***p = 0.049; ****p = 0.01. Scale bar is 50 µm.

To capture the mechanical evolution of the neural plate during the entire procedure of NTC, we conducted time-lapse mechanical mapping of *ex ovo* cultured embryos. The embryo was continuously cultured for more than 14 h (Supplementary Fig. S3), within which the time-lapse Brillouin image of the neural plate cross-section was acquired at the hindbrain/cervical region (Fig. 3a) at the third somite pair along the developing neural plate (Supplementary Fig. S3). The results show that the averaged Brillouin shift of the neural plate continuously increases with culturing time (Fig. 3b), which is consistent with the result of *in ovo* cultured embryos. Repeat experiments (N = 9) suggest the increase in Brillouin shift of the neural plate during NTC is a common phenomenon for chick embryos (Fig. 3d). Specifically, the Brillouin shift of the neural plate increased significantly from HH 8- to HH 9 and had minor changes afterward. At the endpoint of *ex ovo* culture (HH 10), the neural plates have an average Brillouin shift of 6.336 GHz, which is 0.097 GHz higher than that of the earliest stage (HH 8-), corresponding to the relative increase of ~ 46% in terms of the Young’s modulus. This is consistent with the result of *in ovo* cultured embryos, confirming that the *ex ovo* culture and laser illumination did not affect the mechanical evolution of the neural plate tissue during embryonic development.

Using tissue mechanics as a contrast mechanism in Brillouin imaging, we can also quantify the morphological changes of the neural plate during NTC. Here, we measured the averaged thickness of the two sites that is the middle of the distance between the median hinge point and the neural tips (Fig. 3c). Consistent with published literatures^2,33^, we observed four-fold thickening of the neural plate from HH 8- (~ 13 µm) to HH 9 (~ 52 µm) (Fig. 3e). Intriguingly, we found that the tissue thickening and the increase in tissue modulus exhibit very similar trends during the procedure of NTC. We then plotted the Brillouin shift against the thickness for all the *ex ovo* cultured embryos and found a strong correlation ($p<1\times {10}^{-6}$) between the increase in Brillouin shift and the tissue thickening (Fig. 3f). This data suggests that the increase in tissue longitudinal modulus and tissue thickening are probably temporally coordinated events during NTC.

### Increase in tissue modulus and tissue bending are concurrent during NTC

**Figure 4:**
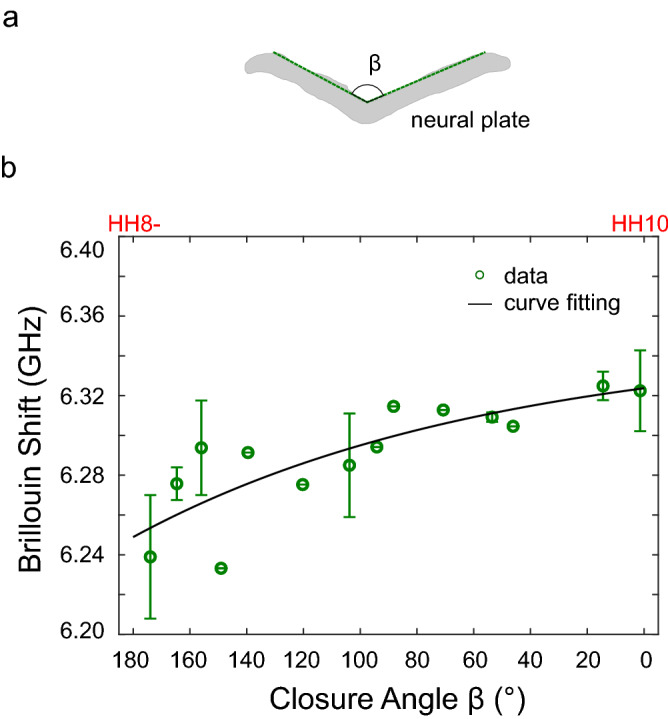
Increase in Brillouin Shift is correlated with tissue bending for *ex ovo* cultured embryos. (**a**) Definition of the closure angle. (**b**) Closure angle is correlated with average Brillouin shift of neural plate. Data from embryos in developmental stages of HH 8- to HH 10 are collected. $\beta =180^\circ$ and 0° represents the neural plate is flat and fully closed, respectively. Embryos are distributed into each 10° interval based on the closure angle. Data point represents the average value of the embryos within the same interval. Error bar represents the standard deviation. Solid curve is fitting result of an empirical function.

NTC is a complex biomechanical process of tissue shaping and patterning that are driven by force and mechanical properties of the tissue. Therefore, it is fundamentally necessary to understand the relationship between tissue mechanics and geometry. Here, we empirically observed how the increase in tissue modulus is coordinated to the geometric change of the neural plate. According to the Brillouin images, we defined the closure angle $\beta$ as the intersection of the left- and right- sides of the neural plate at the median hinge point (Fig. 4a). Based on the definition, $\beta =180^\circ$ and 0° represents the neural plate is flat and fully closed, respectively. Next, we distributed *ex ovo* cultured embryos (developmental stages between HH8- and HH10) into each 10º interval and calculated the averaged values for any interval having multiple embryos. We then plotted the Brillouin shift ${\omega }_{B}$ against the closure angle $\beta$ (Fig. 4b). The data can be well fitted by a simple empirical curve ${\omega }_{B}=A\cdot \mathrm{exp}\left(B\cdot \beta \right)+C$, with fitted parameters $A=-0.024, B=7.85\times {10}^{-3},$ $C=6.348$, and the fitting coefficient ${R}^{2}=0.67$. This positive correlation suggests that the increase in tissue modulus is probably synchronized with the bending of the neural plate during the procedure of NTC. Note that this analysis did not consider the embryos earlier than HH 8- or later than HH 10, at which stages the tissue mechanics might be different for the same closure angle (180° or 0°). Since Brillouin microscope has a confocal configuration, the scattering angle will not be affected by the bending of the neural plate; thus, we excluded the potential artifacts introduced by tissue bending to the observed increase in Brillouin shift. Further investigation is needed to understand the underlying biological mechanism that coordinates the increase in tissue modulus and bending.

## Discussion

Here, we developed an optical modality for time-lapse mechanical mapping of live chick embryos. This modality is based on the combination of a confocal Brillouin microscope and a modified *ex ovo* culturing system, which has subcellular resolution and sufficient mechanical sensitivity. Different from conventional techniques for mechanical testing, our method used a focused laser beam to quantify the tissue mechanics, making it non-contact, non-invasive, and label free. We confirmed that the *ex ovo* culture and laser illumination did not disturb the development of the embryo. We demonstrated the feasibility of this technique by acquiring 2D mechanical images of the neural plate in situ as the embryo experienced neurulation. We found that the neural plate tissue experienced a continuous increase in tissue modulus during NTC. Tissue stiffening during neurulation has been reported in other species previously. For example, the elastic modulus of the neuroepithelium of axolotl embryos increased by one fold from stage 13 to stage 15^19^. In addition, the dorsal neural tissue in early Xenopus embryo has been observed to increase almost 4 folds from stage 11.5 to stage 21^11^. Recently, the preliminary studies on mouse embryo suggest as large as an 80% increase of tissue modulus during neurulation by Brillouin microscopy^31,34^. Although these data are not directly comparable to this work due to the differences in species and/or the used techniques, the similar trend observed in chick embryos suggests that tissue biomechanics may play an important role during NTC across species. The increase in tissue modulus is likely caused by the increased cell density and/or the accumulation of actomyosin contractility^10,11,15^, which should be investigated in the future work. Beyond that, we observed the increase in tissue modulus is strongly correlated with the tissue thickening and bending.

Neurulation is a complex process involving cellular, molecular, and biomechanical activities^9,36^. While the genetic regulation and biochemical signaling have been extensively investigated, the biomechanical mechanism is less explored, and the underlying linkage between microscopic cellular/molecular activities and macroscopic morphogenesis is mostly unknown^5^. Since our all-optical technique can directly quantify the 2D/3D tissue mechanics within intact live embryos, it can potentially open up new opportunities to better understand the role of biomechanical mechanisms in the procedure of NTC. For example, a couple of crucial cellular activities including convergent extension^37^, apical constriction^38^, and interkinetic nuclear migration^39,40^ may together coordinate the observed increase in Brillouin shift, thickening, and bending. The subcellular resolution of the Brillouin microscope will allow researchers to further investigate the role of these cellular behaviors in regulating tissue biomechanics. On the other hand, the mechanical cues can guide cell behaviors and cell fates through mechanotransduction during embryonic development^36,41^; thus, the technique can help understand the interaction between biochemical signaling and biomechanical cues. In addition, the closure of the neural tube is physically driven by both the generated force and the mechanical resistance of the tissue^10,12,42^. The *in situ* quantification of the mechanical properties can help decouple the roles of the force and the tissue mechanics and thus allow better elucidation of the biomechanical interactions. Furthermore, the computational modeling is a powerful tool to understand the mechanism of morphogenesis^43–45^. The time-lapse mechanical images of the neural plate tissue acquired by our technique can provide new input data for the simulation of neurulation.

Neural tube defects (NTDs) are among the commonest human birth diseases and regulated by both genetic and environmental factors^46,47^. On the tissue level, NTDs arise from the physical failure of the neural tube due to the abnormal interaction of force generation and the mechanical properties of embryonic tissue. Recent work suggests that the NTD induced by gene mutation is associated with altered tissue biomechanics^10^. Therefore, a quantitative tool for measuring tissue mechanics should allow researchers to attribute different NTDs to specific dysregulation of cellular mechanisms that cause the failure of the tissue closure, which could bridge the gap between genetic/environmental factors and tissue biomechanics and help the prevention of the diseases.

It is worth noting that, by definition, the high-frequency longitudinal modulus measured by the Brillouin technique is different from the low-frequency or quasi-static Young’s modulus measured by conventional methods such as AFM. However, for many biological materials, it is found that the two moduli change in the same direction in response to biological activities^48,49^. Therefore, with a careful calibration for specific materials, one might interpret Brillouin data in terms of Young’s modulus. In this work, we used the Brillouin shift to estimate the relative change of the tissue modulus by assuming the ratio of density and refractive index ($\rho /{n}^{2}$) is constant. To directly quantify the Brillouin-derived modulus, Brillouin microscopy can be combined with other techniques that can measure the density and/or refractive index^50^. As the image depth increases, the strength of the Brillouin signal will drop depending on the transparency of the tissue. For chick embryos, the maximum penetration depth of our instrument is about 200 µm. Further improvement can be achieved by using a laser source with a longer wavelength or a wavefront correction technique based on adaptive optics^51^. The observed lower Brillouin shift at the boundary of the neural plate in Figs. 2 and 3 is probably an artifact of the experiment. In the confocal configuration, the Brillouin shift at each pixel is the average of all Brillouin signals from the focused beam spot (voxel). At the boundary of the neural plate, since the beam spot is partly filled with surrounding medium and/or mesoderm that has a lower Brillouin shift, the averaging effect will cause a decrease of the ultimate Brillouin shift. This effect can be mitigated by using a high NA objective lens along with data postprocessing^35^.

## Methods

### Eggs and *in ovo* culture

Fertilized white leghorn eggs were purchased from the poultry farm of the University of Connecticut. For *in ovo* culturing, eggs were incubated at 37 °C under high humidity. Incubation hours follows Hamburger-Hamilton (HH) staging (i.e. 26–29 h of incubation to obtain HH-4 embryo)^52^. All experiments carried out in this study were in accordance with relevant guidelines and regulations, and the experimental protocols were approved by the Biosafety office of the University of Maryland. No live vertebrates were used in this study.

### Modified *ex ovo* culture of chick embryo

The *ex ovo* culture protocol was derived from Chapman et al. and Schmitz et al.^53,54^ with modifications for adapting to the Brillouin microscope. A 35 mm glass bottom dish with a 20 mm micro-well (Cellvis, D35-20-0-N) was used for *ex ovo* culture. A 1-inch metallic ring (Thorlabs, SM1RR) was covered with a single-layer Parafilm and a 1 cm ellipse hole was cut into the center and placed over the micro-well (inner bottom well of dish). To perform *ex ovo* culture, we used the filter paper carrier method to hold the blastoderm and vitelline membrane under tension to mimic the situation of *in ovo* culturing. The pre-cultured embryo around HH 4 was collected from the egg and then placed dorsal side down onto a culture dish filled with thin albumin harvested from the egg (culture medium). Next, the dish was placed into an on-stage incubator (Warner Instruments, SA-20PLIXR-AL) for continuous culturing. To ensure the ambient temperature of the embryo is about 37 °C, the heater (Warner Instruments, TC-344C) of the on-state incubator was set to 39 ± 0.2 °C considering the heat dissipation from the underside of the stage which is open to the objective lens.

### Brillouin light scattering

Spontaneous Brillouin light scattering is the interactions between the incident light and the inherent acoustic phonons inside a sample. The result of this interaction introduces a frequency shift (Brillouin shift) to the outgoing scattered light. The Brillouin shift ${\omega }_{B}$ is defined as ${\omega }_{B}=2n/\lambda \cdot \sqrt{{M}^{^{\prime}}/\rho }\cdot \mathrm{sin}(\theta /2)$, where $n$ is refractive index of the material, $\lambda$ is the laser wavelength, ${M}^{^{\prime}}$ is the longitudinal modulus that quantifies the mechanical properties, $\rho$ is the density, and θ is the collection angle of the scattered light. In our Brillouin microscope, backward scattered light was collected, yielding $\theta =180^\circ$.

### Estimation of Young’s modulus using measured Brillouin shift

For biological materials, an empirical relationship between Young’s modulus $E$ and Brillouin-derived longitudinal modulus ${M}^{^{\prime}}$ has been established: $\mathrm{log}\left({M}^{^{\prime}}\right)=a\cdot \mathrm{log}\left(E\right)+b$, where the parameters $a$ and $b$ are material dependent^24^. Considering the relationship between ${M}^{^{\prime}}$ and Brillouin shift ${\omega }_{B}$, the relative change of Brillouin shift is related to that of Young’s modulus by $\Delta E/E=2/a\cdot \Delta {\omega }_{B}/{\omega }_{B}$. For cells, the calibration against AFM shows $a=0.0671$. Here, we used this calibrated relationship to estimate the relative change of Young’s modulus.

### Time-lapse Brillouin imaging

A confocal Brillouin microscope was used for all experiments. The detail of the instrumentation can be found in our recent report^25^. Briefly, a single mode 660 nm continuous wave laser with power of ~ 30 mW was used as light source. The laser beam was focused into the sample by an objective lens (Olympus, 40 × /0.6 NA) installed on an inverted microscope (Olympus, IX81), which yields a spot size of 0.7 μm × 0.7 μm × 2.6 μm. The backward scattered Brillouin signal was collected by the same objective and analyzed by a two-stage VIPA (Light Machinery, 15 GHz FSR) based spectrometer, and the Brillouin spectrum was recorded by an EMCCD camera (Andor, iXon 897) with an exposure time of 0.05 s. 2D Brillouin images were acquired by scanning the sample using a motorized stage (step size: 0.5 µm). The cross-section perpendicular to the anterior–posterior body axis was mapped by Brillouin microscope, and the averaged Brillouin shift of the neural plate region was used to represent the mechanical properties of the tissue. The only contrast of a Brillouin image is from the difference in the Brillouin shift of pixels. The Brillouin shift of the thin albumin culture medium is very close to that of the neural plate for early-stage embryos, making it difficult to identify the boundary of neural plate tissue in the Brillouin image. To solve this issue, right before acquiring each Brillouin image, the embryo was temporarily transferred onto a different culture dish filled with Ringer’s solution (Thermo Scientific, BR0052G) which has much lower Brillouin shift and thus providing a good contrast. As soon as the Brillouin measurement is done, the embryo was transferred back onto thin albumin culture medium for continuous development. To acquire the bright-field images, a low magnification objective lens (Olympus, 4x/0.1) and a CMOS camera (Andor Neo) were used when the embryo was in the *ex ovo* culturing dish.

### Data acquisition and analysis

A home built LabView (National Instruments, ver.2021) acquisition program was used to acquire both bright-field images and the Brillouin spectra. For calibration of the spectrometer, Brillouin spectra of materials (water and methanol) with known Brillouin shifts were recorded and used to calculate the free spectral range and the pixel-to-frequency convention ratio. The Brillouin shift of each pixel was obtained by fitting the Brillouin spectrum to a Lorentzian function using MATLAB (MathWorks, R2021b). 2D Brillouin images were reconstructed from the pixel vector. Sample size was chosen based on previous experience and to be reasonably large to demonstrate the feasibility of the technique.

## Supplementary Information

Supplementary Figures.

## Data Availability

All data supporting the findings of this study are available within the paper and its Supplementary Information files.
