# Supplementary material for: Time-lapse mechanical imaging of neural tube closure in live embryo using Brillouin microscopy
Source: Sci Rep. 2023 Jan 6;13:263. doi: 10.1038/s41598-023-27456-z (PMC9823106; doi:10.1038/s41598-023-27456-z)
Supplement: Supplementary file 1 — Supplementary Figures. [file 41598_2023_27456_MOESM1_ESM.pdf]

Supplementary Information for

Time-lapse mechanical imaging of neural tube closure in live embryo using Brillouin microscopy

Chenchen Handler[1], Giuliano Scarcelli[1] and Jitao Zhang[2,*]

[1] Fischell Department of Bioengineering, A. James Clark School of Engineering, University of Maryland, College Park, MD 20742, USA

[2] Department of Biomedical Engineering, Wayne State University, Detroit, MI 48201, USA

[*] Corresponding author: zhang4@wayne.edu

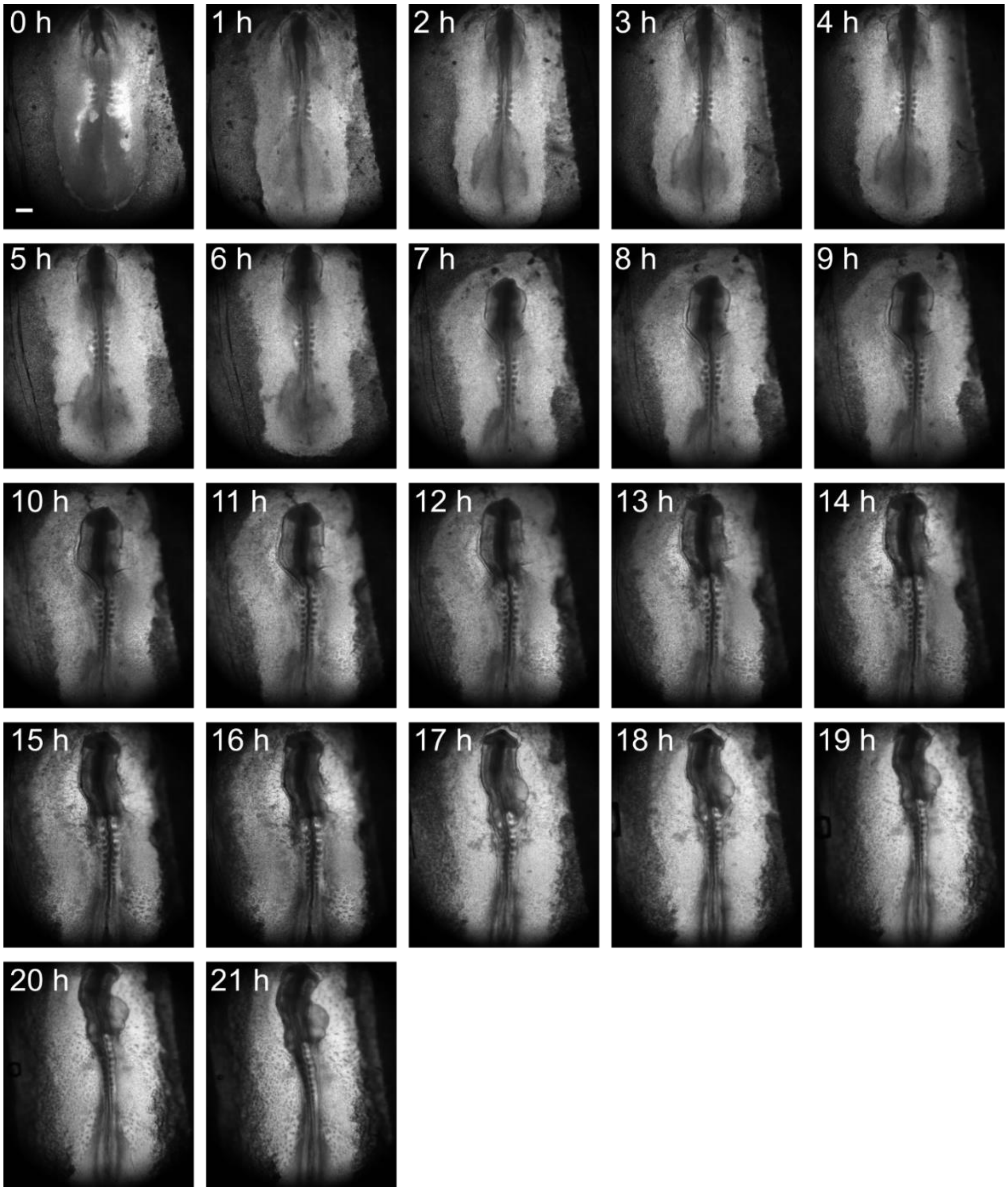

Supplemental Figure S1. Brightfield time-lapse imaging of *ex ovo* cultured embryo without Brillouin measurement. Representative of continuous *ex ovo* development on thin albumin without laser illumination for 21 hrs. An HH8 embryo is extracted for *ex ovo* culture after 29 hrs of *in ovo* incubation (0 h). Embryo is identified as HH13+ at 21 h of *ex ovo* incubation (21 h) with a total incubation time of 50 hrs. Embryo develops according to the HH stages with identifiable markers at each stage. Each image taken 1 h apart. Scale bar is 300 µm.

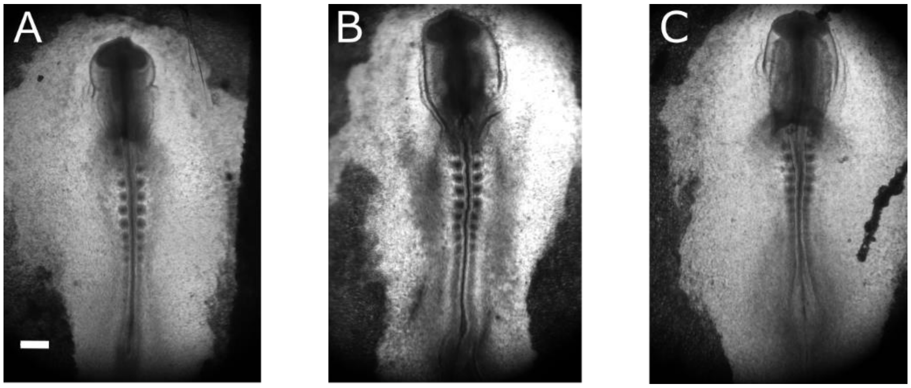

Supplemental Figure S2. Comparison of all incubation and experimental methods reveal similar development. (A) HH 10 embryo after 39 hrs of continuous culture (30 hrs *in ovo* + 9 hrs *ex ovo*) on thin albumin without laser illumination. (B) HH 10 embryo after 38 hrs of time-lapse *ex ovo* culture (30 hrs *in ovo* + 8 hrs *ex ovo*) including transfer onto Ringer's solution and exposure to laser illumination during Brillouin acquisition. (C) HH 10 embryo after 38 hrs of *in ovo* culture extracted for Brillouin mapping. All three embryos experienced similar development time following HH stages and display similar morphology with different methods of incubation and experimental setting. Scale bar is 300 µm.

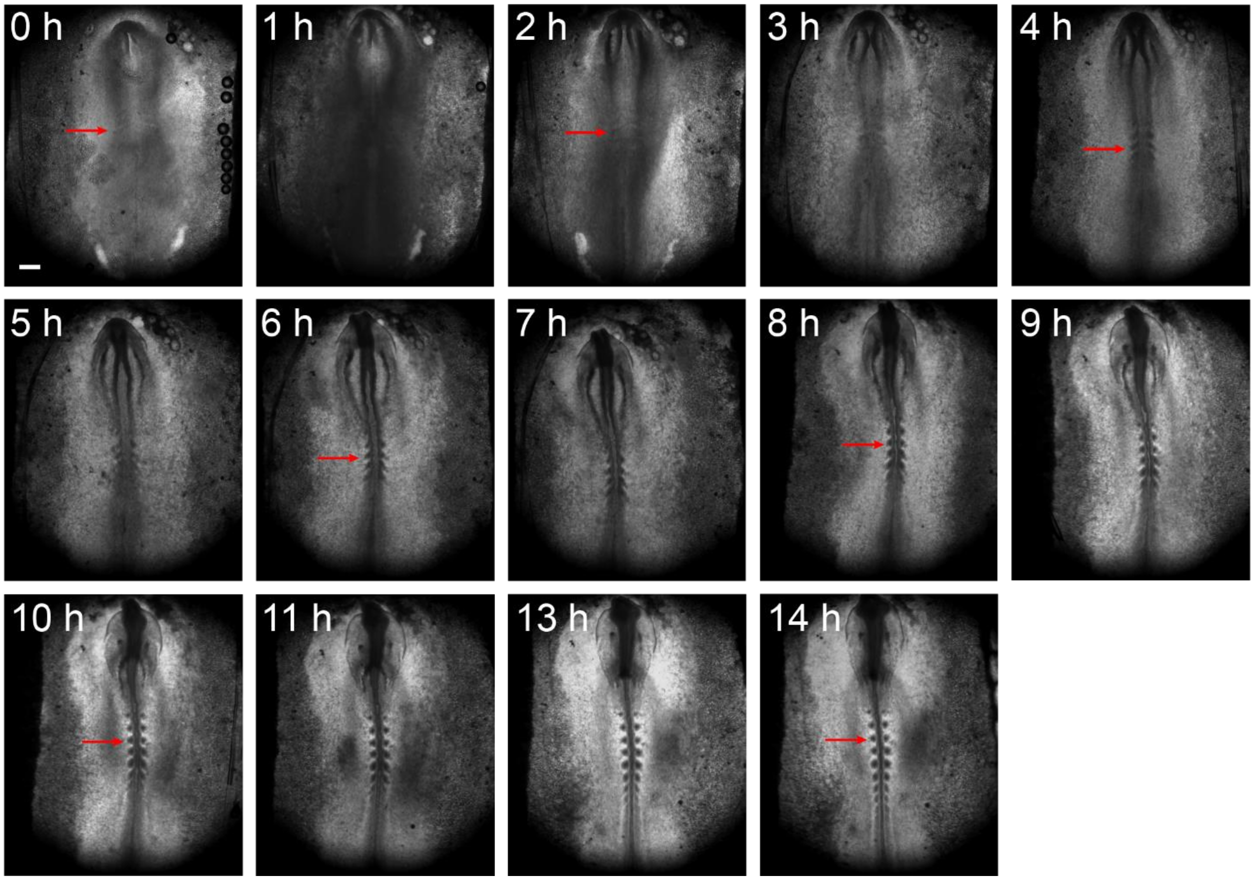

Supplemental Figure S3. Brightfield time-lapse Brillouin imaging of *ex ovo* cultured embryo. Representative of 14 hrs of *ex ovo* development during Brillouin imaging, including transfer onto Ringer's solution and exposure to laser illumination. An HH 8 embryo was extracted for *ex ovo* culture and time-lapse Brillouin imaging after 26 hrs *in ovo* incubation (0 h) and developed to an HH 10 embryo after 14 hrs of *ex ovo* incubation (14 h). Brillouin acquisition was conducted every 2 hrs with the initial acquisition at 0 h (HH8). This is to allow the embryo to develop on thin albumin between acquisition. Each image taken 1 h apart, 12 h not displayed. Scale bar is 300 µm. The red arrow indicates the location of Brillouin measurement.
